# Silencing of uncoupling protein 2 by small interfering RNA aggravates mitochondrial dysfunction in cardiomyocytes under septic conditions

**DOI:** 10.3892/ijmm.2015.2177

**Published:** 2015-04-09

**Authors:** GUILANG ZHENG, JUANJUAN LYU, SHU LIU, JINDA HUANG, CUI LIU, DAN XIANG, MEIYAN XIE, QIYI ZENG

**Affiliations:** Department of Pediatrics, Zhujiang Hospital, Southern Medical University, Guangzhou, Guangdong 510282, P.R. China

**Keywords:** uncoupling protein 2, mitochondrial DNA, mito chondrial function, small interfering RNA, sepsis, H9C2, reactive oxygen species, mitochondrial membrane potential

## Abstract

Uncoupling protein 2 (UCP2) regulates the production of mitochondrial reactive oxygen species (ROS) and cellular energy transduction under physiological or pathological conditions. In this study, we aimed to determine whether mitochondrial UCP2 plays a protective role in cardiomyocytes under septic conditions. In order to mimic the septic condition, rat embryonic cardiomyoblast-derived H9C2 cells were cultured in the presence of lipopolysaccharide (LPS) plus peptidoglycan G (PepG) and small interfering RNA (siRNA) against UCP2 (siUCP2) was used to suppress UCP2 expression. Reverse transcription quantitative-polymerase chain reaction (RT-qPCR), western blot analysis, transmission electron microscopy (TEM), confocal microscopy and flow cytometry (FCM) were used to detect the mRNA levels, protein levels, mitochondrial morphology and mitochondrial membrane potential (MMP or ΔΨm) in qualitative and quantitative analyses, respectively. Indicators of cell damage [lactate dehydrogenase (LDH), creatine kinase (CK), interleukin (IL)-6 and tumor necrosis factor (TNF)-α in the culture supernatant] and mitochondrial function [ROS, adenosine triphosphate (ATP) and mitochondrial DNA (mtDNA)] were detected. Sepsis enhanced the mRNA and protein expression of UCP2 in the H9C2 cells, damaged the mitochondrial ultrastructure, increased the forward scatter (FSC)/side scatter (SSC) ratio, increased the CK, LDH, TNF-α and IL-6 levels, and lead to the dissipation of MMP, as well as the overproduction of ROS; in addition, the induction of sepsis led to a decrease in ATP levels and the deletion of mtDNA. The silencing of UCP2 aggravated H9C2 cell damage and mitochondrial dysfunction. In conclusion, our data demonstrate that mitochondrial morphology and funtion are damaged in cardiomyocytes under septic conditions, while the silencing of UCP2 using siRNA aggravated this process, indicating that UCP2 may play a protective role in cardiomyocytes under septic conditions.

## Introduction

Sepsis is a systemic inflammatory response syndrome (SIRS) caused by probable or documented infection, which continues to pose serious clinical challenges. Sepsis affects individuals of all ages and is the leading cause of morbidity and mortality for critically ill patients (1). Sepsis-induced myocardial dysfunction (SIMD), one of the main predictors of morbidity and mortality associated with sepsis, is present in >40% of cases of sepsis (2). SIMD increases the mortality rate of sepsis by up to 70% (3). A number of studies on both patients and experimental animals (4–6) have indicated that mitochondrial dysfunction seems to be related to the severity and prognosis of sepsis. The heart is rich in mitochondria, and thus the role of mitochondrial damage in SIMD has received much attention. Previous studies have indicated that multiple aspects of mitochondrial dysfunction contribute to SIMD, such as the overproduction of reactive oxygen species (ROS), the altered generation of adenosine triphosphate (ATP) and the disruption of mitochondrial membrane potential (MMP or ΔΨm) (78)

Uncoupling proteins (UCPs) may constitute a vital link between ATP and ROS production (9,10); they are inner mitochondrial membrane proteins that disperse the mitochondrial proton gradient by translocating H^+^ across the inner membrane. Of the 5 UCP homologues, uncoupling protein 2 (UCP2) is ubiquitously expressed; for example, it is expressed in the liver, brain, pancreas, adipose tissue, immune cells, spleen, kidneys and heart. *In vitro* and *in vivo*, by modulating MMP, as well as the ATP and ROS levels, the upregulation of UCP2 plays a neuroprotective role (11–13), while UCP2 knockout mice present with increased mitochondrial ROS production (9). In addition, UCP2 is involved in the regulation of other physiological or pathological events, such as in the formation of atherosclerotic plaque, food intake and metabolic diseases (14). Given its central role in regulating mitochondrial ROS production and cellular energy transduction, we hypothesized that UCP2 may play a protective role in sepsis. To the best of our knowledge, the possible role of UCP2 in sepsis has received little attention. However, some scholars have found that UCP2 deficiency provides protection in acute liver failure induced by endotoxemic stress (15) and in the pathogenesis of experimental leishmaniosis (16). Nonetheless, whether UCP2 plays a protective role in sepsis needs to be determined.

In early experiments, we found high levels of UCP2 gene and protein expression in septic myocardial tissue (unpublished data), which is consistent with the findings of other studies (17,18). However, the exact role of UCP2 in myocardial cells under septic conditions remains to be determined. It remains unclear as to whether UCP2 plays a protective role in myocardial cells under septic conditions by regulating MMP and the generation of mitochondrial ROS. We hypothesized that UCP2 may regulate MMP and mitochondrial function under septic conditions. In this study, we measured the levels of ROS and ATP production, as well as the extent of MMP and other relative indicators following the silencing of UCP2 by RNA interference technology in H9C2 cardiomyocytes under septic conditions.

## Materials and methods

### Small interfering RNA (siRNA) transfection

Two siRNAs against UCP2 (siUCP2) and negative control siRNA (ncRNA) were synthesized by Shanghai GenePharma Co., Ltd. (Shanghai, China). The sequences of siRNA1 were as follows: forward, 5′-GCA CUG UCG AAG CCU ACA A dTdT-3′ and reverse, 5′-UUG UAG GCU UCG ACA GUG C dTdT-3′. The sequences of siRNA2 were forward, 5′-CCU CAU GAC AGA CGA CCU C dTdT-3′ and reverse, 5′-GAG GUC GUC UGU CAU GAG G dTdT-3′. The sequences of ncRNA were forward, 5′-UUC UCC GAA CGU GUC ACG UTT-3′ and reverse, 5′-ACG UGA CAC GUU CGG AGA ATT-3′. The siRNAs were transfected using Lipofectamine 2000 (Invitrogen Life Technologies, Carlsbad, CA, USA) according to the manufacturer’s instructions. Briefly, siRNA (final concentration 80 nmol/l) and Lipofectamine 2000 were firstly diluted separately in Opti-DMEM medium (Gibco™; Life Technologies, Grand Island, NY, USA) without antibiotics or serum, and incubated together for 20 min. The complexes were then added to the H9C2 cells. After 6 h of incubation, the medium was changed as needed. The silencing efficiency of the 2 siRNAs was tested in experiments of transient RNA interference; the UCP2 transcript was assayed by reverse transcription-quantitative PCR (RT-qPCR) 48 h after infection. siRNA2, which showed the most prominent silencing effect (69% knockdown efficiency of mRNA) was used for the subsequent experiments. For further experiments, the H9C2 cells were cultured following transfection.

### Cell culture and treatment

Rat embryonic cardiomyo-blast-derived H9C2 cells were purchased from the Typical Culture Preservation Commission Cell Bank, Chinese Academy of Sciences (Shanghai, China). H9C2 cells mimic most of the characteristics of adult cardiomyocytes and this is an ideal cell line with which to explore the role of UCP2 in the septic myocardium in a cell culture system. The cells were cultured in DMEM (Gibco-BRL, Beijing, China) supplemented with 10% fetal calf serum and 5% CO_2_ at 37°C. The H9C2 cells were passaged regularly and subcultured to 75% confluence prior to use in the experiments. In order to simulate sepsis, some cells were cultured in the presence of 2 *μ*g/ml lipopolysaccharide (LPS, from *Escherichia coli* O111:B4; Sigma-Aldrich, St. Louis, MO, USA) plus 20 *μ*g/ml peptidoglycan G (PepG, from *Micrococcus luteus;* Sigma-Aldrich). The experimental design consisted the following 4 groups: i) the control group, cells were treated with saline only; ii) the LPS/PepG group, cells treated with LPS and PepG as described above; iii) the LPS/PepG + siRNA group, cells transfected with siRNA2 and 24 h later treated with LPS plus PepG as described above; and iv) the LPS/PepG + ncRNA group, cells transfected with ncRNA and 24 h later treated with LPS plus PepG as described above. Further experiments were carried out 24 h following stimulation with LPS plus PepG.

### RT-qPCR

To examine the mRNA levels of UCP2, total RNA was extracted from the H9C2 cells using TRIzol reagent (Invitrogen Life Technologies) and then reverse transcribed and synthesized into cDNA using RT-PCR kits (Toyobo Co., Ltd., Osaka, Japan). RT-PCR amplification reaction was performed in a volume of 10 *μ*l containing 0.25 *μ*l forward/reverse primers, *5 μ*l SYBP-Green PCR Master Mix and 4 *μ*l cDNA. PCR was performed for 45 cycles of 5 min at 95°C, 15 sec at 95°C, and 30 sec at 60°C. The threshold cycle (Ct) was obtained from triplicate samples and averaged. Calculations were based on the ‘ΔΔCt method’ using the equation R (ratio) = 2^−ΔΔCt^ and standardized by the housekeeping gene, 18s RNA. The specific primers for UCP2 (Shanghai GenePharma Co., Ltd.) were: forward, 5′-GGG CAC CTG TGG TGC TAC CTG-3′ and reverse, 5′-ATG AGC TTT GCC TCC GTC CGC-3′; and those for 18s RNA were: forward, 5′-CCA TCC AAT CGG TAG TAG C-3′ and reverse, 5′-GTA ATG GCG GGT CAT AAG-3′.

### Western blot analysis

The H9C2 cells were lysed in 2X SDS sample buffer and the protein concentrations in the super-natants were measured by BCA Protein assay. An equal amount of protein (30 mg) from each sample was subjected to 12–15% SDS-PAGE gels and then transferred onto PVDF filter membranes (Millipore, Billerica, MA, USA). The membranes were blocked with 5% (w/v) non-fat dried skimmed milk powder in wash buffer (Tris-buffered saline/1% Tween-20) for 1 h and subsequently incubated with primary antibodies overnight at a dilution recommended by the suppliers. The membranes were washed 3 times with wash buffer and then incubated with corresponding horseradish peroxidase-conjugated secondary antibodies. The protein signal was developed using ECL substrate (Beyotime Institute of Biotechnology, Jiangsu, China) according to the manufacturer’s instructions. The immunoreactive protein bands were visualized using the In-Vivo Imaging System F (Eastman Kodak Co., Rochester, NY, USA). The band intensity was quantified using of Gel-Pro Analyzer 4.0 software.

### Measurement of lactate dehydrogenase (LDH) and creatine kinase (CK) levels

Forty-eight hours after cell treatment, the culture supernatant was collected for the subsequent measurement of CK and LDH levels. The release of the cytosolic enzymes, CK and LDH, indicators of cytotoxicity, reflected a loss of membrane integrity in the damaged cells and was detected by colorimetric assay. CK and LDH activity was measured using commercially available kits (Nanjing Jiancheng Bioengineering Institute, Jiangsu, China), according to the manufacturer’s instructions. Absorbance was respectively read at 660 and 440 nm on a multifunctional microplate reader (SpectraMax M5/M5e; Molecular Devices, Sunnyvale, CA, USA). The release of CK and LDH was calculated relative to the percentage of the control group.

### Enzyme-linked immunosorbent assay (ELISA) for the detection of interleukin (IL)-6 and tumor necrosis factor (TNF)-α

Forty-eight hours after cell treatment, the culture supernatant was collected for the subsequent measurement of IL-6 and TNF-α expression levels. The culture supernatants were measured using commercially available ELISA kits (Cusabio Life Science, Wuhan, China). All procedures were performed strictly as per the instructions of the manufacturer. The samples were analyzed in triplicate.

### Degree of mitochondrial swelling

To determine the large amplitude swelling of the H9C2 cells, the isolation of the mitochondria and the cytosol was performed using the Cell Mitochondria Isolation kit (Beyotime Institute of Biotechnology). Mitochondrial fractions were separated by differential centrifugation according to the manufacturer’s instructions. Briefly, the H9C2 cells were incubated in ice-cold mitochondrial lyses buffer for 15 min. The cell suspension was then poured into a glass homogenizer and homogenized for 20 strokes. The homogenate was subjected to centrifugation at 600 × g for 10 min at 4°C to remove the nuclei and unbroken cells. The supernatant was then collected and centrifuged again at 11,000 × g for 15 min at 4°C to obtain the mitochondrial fraction. Samples of mitochondria were dissolved in lysis buffer and subjected to flow cytometry (FCM). The size [(forward scatter (FSC)] and structure [side scatter (SSC)] of the mitochondria was determined. The degree of mitochondrial swelling was quantified as the FSC/SSC ratio, as previously described (19).

### Transmission electron microscopy (TEM)

The H9C2 cells were col lected and fixed in a solution containing 3.0% formaldehyde, 1.5% glutaraldehyde in 100 mM cacodylate containing 2.5% sucrose (pH 7.4). The H9C2 cells were stained with 4% aqueous uranyl acetate, dehydrated, infiltrated and embedded in epoxyresin. Ultrathin sections (80 nm) were cut and imaged using a Hitachi transmission electron microscope (H-7500; Hitachi, Tokyo, Japan).

### MMP (or ΔΨm)

ΔΨm was assessed using a laser scanning confocal microscope (LSCM, FV10i-W; Olympus Corp., Tokyo, Japan) and a flow cytometer (BD FACSAria; BD Biosciences, Franklin Lakes, NJ, USA) with 5,5′,6,6′-tetrachloro-1,1′,3,3′-tetraethylbenzimidazole-carbocyanide iodine (JC-1; Beyotime Institute of Biotechnology) staining. The H9C2 cells were stained with JC-1 for 20 min at 37°C after 24 h of incubation with LPS/PepG. Cells on a 35-mm confocal special dish were scanned using an LSCM, and mitochondrial suspension was detected by FCM. Fluorescence was read at 488 nm excitation and 530 nm emission for green, and at 540 nm excitation and 590 nm emission for red. Cells treated with 10 *μ*M carbonyl cyanide m-chlorophenylhydrazone (CCCP) which can cause the dissipation of ΔΨm were used as positive controls. The ratio of aggregated JC-1 (red fluorescence) and monomeric JC-1 (green fluorescence) represented the ΔΨm of H9C2 the cells.

### Assay of intracellular ROS

The production of ROS in the H9C2 cells was fluorometrically monitored using the non-fluorescent probe, 2′,7′-dichlorofluorescein diacetate (DCFH-DA) (Beyotime Institute of Biotechnology). DCFH-DA passively diffuses into cells and is deacetylated, changing into the fluorescent compound, 2′,7′-dichlorofluorescein (DCFH). DCFH reacts with ROS to form the fluorescent product, DCF, which is trapped inside the cells. Cells in 6-well culture dishes were trypsinized, and collected by centrifugation. DCFH-DA, diluted to a final concentration of 10 *μ*M with DMEM, was added to the H9C2 cells followed by incubation at 37°C for 20 min. Following treatment with DCFH-DA, the H9C2 cells were washed 3 times with PBS. DCF fluorescence was then read using a multifunctional microplate reader (SpectraMax M5/M5e; Molecular Devices) at an excitation wavelength of 488 nm and an emission wavelength of 525 nm. The increase in the value of the levels of ROS was expressed as a percentage of the control. To visually observe the changes in ROS production, fluorescence images were acquired using a fluorescence microscope with 450–490 nm (excitation) and 520 nm (emission) filters.

### Detection of cellular ATP levels

The cellular amount of ATP was measured using a firefly luciferase-based ATP assay kit (Beyotime Institute of Biotechnology) according to the manufacturer’s instructions. Briefly, after 48 h of treatment, the H9C2 cells were treated with lysis buffer and centrifuged at 12,000 × g for 8 min. In 24-well plates, 50 *μ*l of each supernatant were mixed with 100 *μ*l ATP detection working dilution. Luminance [in relative luminance units (RLU)] was measured using a multifunctional microplate reader (SpectraMax M5/M5e; Molecular Devices). A standard curve of the ATP concentration was prepared from a known amount (0.01–10 *μ*M) and the protein concentration in each group was detected using the Bradford protein assay (Beyotime Institute of Biotechnology, Jiangsu, China). The ATP levels were expressed as nmol/mg protein.

### Determination of mitochondrial DNA (mtDNA) copy number by real-time PCR

Total genomic DNA was extracted using a DNA extraction kit (Qiagen, Hilden, Germany), and the DNA concentration was measured by optical density. The mtDNA copy number was obtained according to the manufacturer’s instructions (AceQ™ qPCR SYBR^®^-Green Master Mix; Promega Biotech Co., Ltd, Beijing, China). Briefly, DNA primers were designed to detect Cytb and 18s RNA at a maximum amplicon length of 150 bp: 18s RNA forward, 5′-GTAAGTGCGGGTCATAAG-3′ and reverse, 5′-CCATCCAATCGGTAGTAGC-3′; Cytb forward, 5′-GTCGAATGAATTTGAGGGGG-3′ and reverse, 5′-GAGGAGGTGAACGATTGCTAGG-3′. The PCR reaction mixture contained 2X SYBR-Green PCR Master Mix 10 *μ*l, each primer 0.5 *μ*l, and total genomic DNA 4.0 *μ*l, and dH_2_O 5.0 *μ*l. The real-time PCR conditions were 5 min at 95°C and followed by 40 cycles of 15 sec at 95°C and 34 sec at 60°C. The Ct is the cycle at which the first significant increase in the fluorescence signal is detected. Relative values for Cytb and 18s RNA in the samples were used to obtain the ratio of mtDNA to nuclear DNA (nDNA) in that sample.

### Statistical analysis

The experimental values were obtained from 3 independent experiments with a similar pattern and are expressed as the means ± SD. For the determination of the statistical differences between the control and treatment groups, we used ANOVA, followed by post hoc pairwise comparison (LSD) tests for analysis. Statistical analysis was carried out using the SPSS software package 20.0. Statistical significance was set at P<0.05.

## Results

### Successful transfection of siRNA and knockdown efficiency of siUCP2

**Figure 1:**
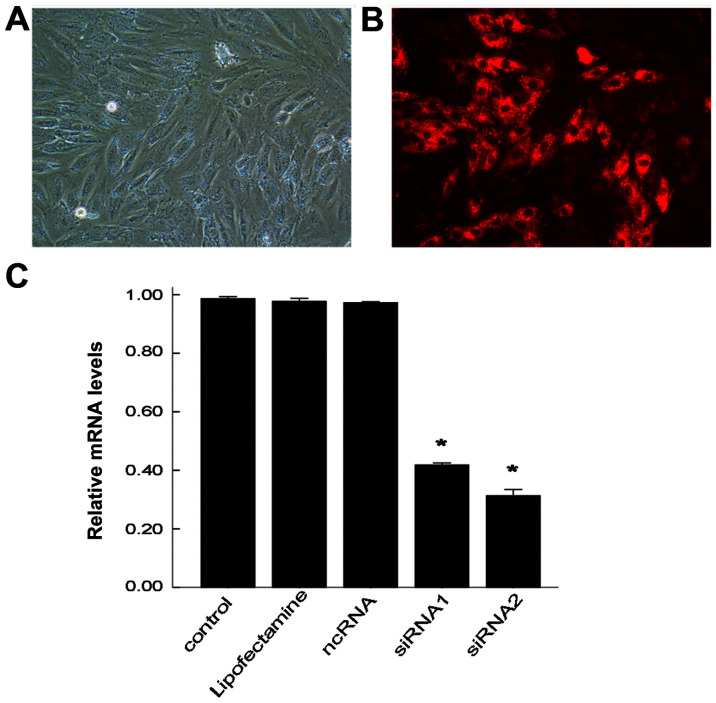
The transfection and knockdown efficiency of siRNA against UCP2 in H9C2 cells. (A and B) H9C2 cells transfected with control siRNA (Cy3) emitted red fluorescence. The transfection efficiency is expressed as the ratio of the number of red fluorescence cells over all cells by fluorescence microscopy (magnification, x20). (C) The knockdown efficiency of siRNA against UCP2 in H9C2 cells. H9C2 cells were left untransfected (control), transfected with Lipofectamine 2000 (no siRNA), transfected with negative control siRNA (ncRNA), or transfected with siRNA1 or 2. After 24 h, the mRNA levels of uncoupling protein 2 (UCP2) were determined by RT-qPCR with normalization to 18s RNA. ^*^P<0.05 vs. control.

The H9C2 cells were successfully transfected with siRNA-cy3 using Lipofectamine 2000 and observed under a fluorescence microscope. The H9C2 cells transfected with siRNA-cy3 all emitted red fluorescence. The transfection efficiency after 24 h was determined through the observation of red fluorescence (Fig. 1A and B). The transfection efficiency was 85% which was evaluated in 500 H9C2 cells randomly.

To examine the function of UCP2 in sepsis, 2 siRNAs against UCP2 were used to specifically suppress UCP2 mRNA expression in this study. The H9C2 cells were either left untransfected (control), transfected with Lipofectamine 2000 (no siRNA), transfected with ncRNA, or with siRNA1 or 2. After 24 h, the mRNA levels of UCP2 were determined by RT-qPCR. The mRNA level of UCP2 was reduced by up to nearly 69% following transfection with siRNA2 and by 59% following transfection with siRNA1 compared with the controls (Fig. 1C). There were no significant changes observed in the UCP2 mRNA level in the cells transfected with Lipofectamine only or with ncRNA compared with the control group. siRNA2 was thus selected for use in further experiments.

### Effects of siRNA on the levels of CK, LDH, IL-6 and TNF-α

**Figure 2:**
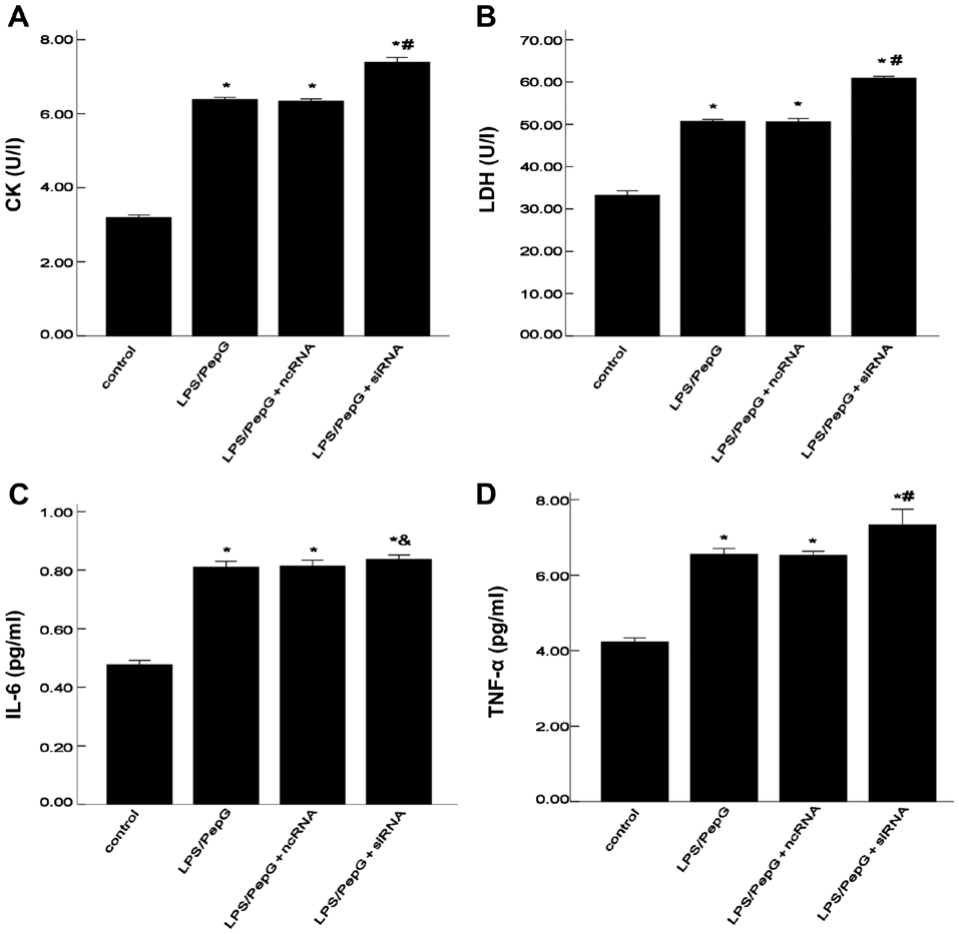
Effects of siRNA on the levels of (A) creatine kinase (CK), (B) lactate dehydrogenase (LDH), (C) interleukin (IL)-6 and (D) tumor necrosis factor (TNF)-α in the culture supernatant of lipopolysaccharide (LPS)/peptidoglycan G (PepG)-treated H9C2 cells. Control, LPS/PepG, LPS/PepG + negative control siRNA (ncRNA), LPS/PepG + siRNA represent the control, LPS/PepG, LPS/PepG + ncRNA and LPS/PepG + siRNA group, respectively. The data are expressed as the means ± SD; n=3 for each group. *P<0.05 vs. control. ^#^P<0.05, ^&^P>0.05 vs. LPS/PepG group.

The CK and LDH levels are biochemical markers for the extent of injury to H9C2 cells (20–22). The increased levels of CK and LDH in the culture supernatant indicate the degree of cell injury. TNF-α and IL-6 are important pro-inflammatory cytokines in sepsis. As shown in Fig. 2, following treatment with LPS/PepG, the levels of CK, LDH, TNF-α and IL-6 in the cell supernatant notably increased compared with those in the control group (no treatment; all P<0.05). In other words, compared with the control group, treatment with LPS/PepG induced a 2-fold increase in CK levels, a 1.5-fold increase in LDH levels, a 1.6-fold increase in IL-6 levels and a 1.5-fold increase in TNF-α levels. The significant increase in CK, LDH, TNF-α and IL-6 levels in the culture supernatant indicated that the H9C2 cell model of sepsis had been successfully constructed. Subsequently, we further explored the effects of siRNA on H9C2 cells under septic conditions. Supernatants from the cells treated with LPS/PepG + siRNA had markedly higher CK, LDH and TNF-α concentrations (all P<0.05; Fig. 2A, B and D) compared with the cells treated with LPS/PepG; however, no statistically significant differences were observed in the IL-6 concentration (P>0.05; Fig. 2C) when compared with the cells treated with LPS/PepG. Compared with the LPS/PepG group, ncRNA had no effect on the CK, LDH, TNF-α and IL-6 levels in the culture supernatant (all P>0.05; Fig. 2).

### UCP2 mRNA and protein levels in H9C2 cells under septic conditions

**Figure 3:**
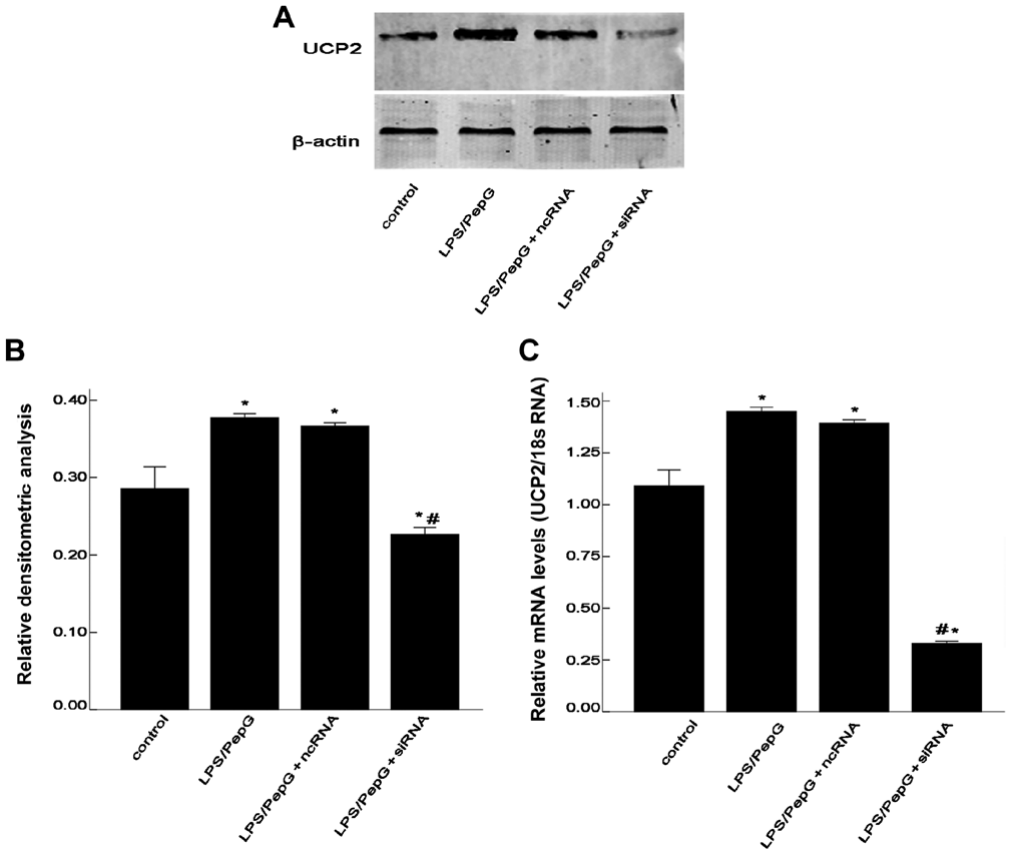
Uncoupling protein 2 (UCP2) mRNA and protein levels in H9C2 cells. Total RNA was extracted and the relative (A and B) protein and (C) mRNA levels of UCP2 were determined by western blot analysis (β-actin was used as a loading control) and RT-qPCR (with normalization to 18s RNA), respectively. Values are the means ± SD; n=3 for each group. ^*^P<0.05 vs. control. ^#^P<0.05 vs. lipopolysaccharide (LPS)/peptidoglycan G (PepG) group.

To determine whether sepsis increases the UCP2 mRNA and protein levels, the H9C2 cells were treated with LPS plus PepG or saline. After 48 h, total RNA was extracted for RT-qPCR and total protein was extracted for western blot analysis. As shown in Fig. 3, the mRNA and protein expression of UCP2 was enhanced by treatment with LPS/PepG. Compared with the controls, sepsis caused a 1.4-fold increase in UCP2 mRNA levels and a 1.3-fold increase in UCP2 protein levels following treatment with LPS/PepG or saline (P<0.05; Fig. 3). To determine the role of UCP2 in cardiomyocytes under septic conditions, the septic H9C2 cells were transfected with siRNA to suppress UCP2 expression. Compared with the LPS/PepG group, siRNA induced a 0.22-fold decrease in UCP2 protein expression and a 0.59-fold decrease in UCP2 mRNA expression (P<0.05; Fig. 3). As shown in Fig. 3, ncRNA had no effect on UCP2 mRNA or protein expression in the H9C2 cells under septic conditions (P>0.05).

### MMP (or ΔΨm) in H9C2 cells under septic conditions

**Figure 4:**
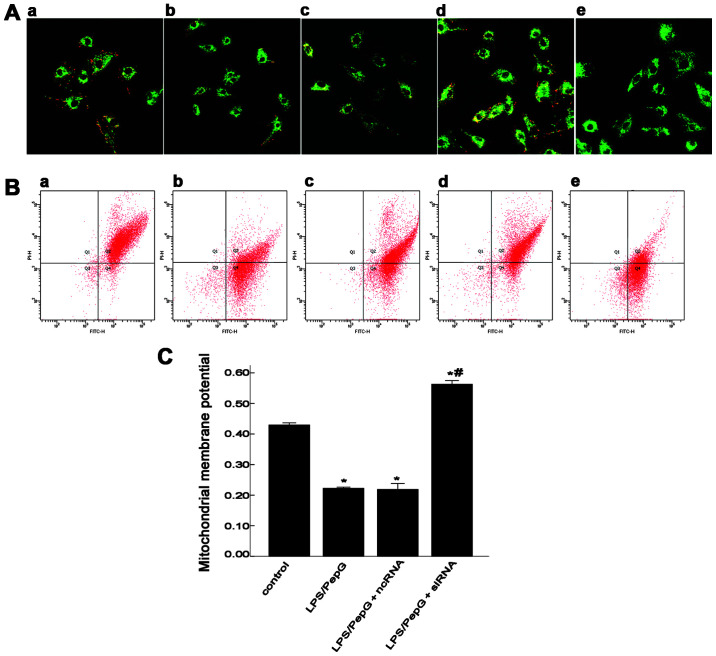
Effect of siRNA on mitochondrial membrane potential (MMP or ΔΨm) in H9C2 cells under septic conditions. (A) Scanning of MMP in H9C2 cells using a confocal microscope. Green fluorescence represents the monomeric form of the JC-1 molecule, which appears in the cytosol after mitochondrial membrane depolarization. Red fluorescence reveals the mitochondrial aggregate form of JC-1, indicating MMP (magnification, x40). (B) Evaluation of MMP in H9C2 cells by flow cytometry (FCM). FITC-H, green; PI-H, red. (A and B) Panel a, control group; panel b, lipopolysaccharide (LPS)/peptidoglycan G (PepG) group; panel c, LPS/PepG + negative control siRNA (ncRNA) group; panel d, LPS/PepG + siRNA group; panel e, carbonyl cyanide m-chlorophenylhydrazone (CCCP)-treated cells. (C) The ratio of the red over the green fluorescence intensity by FCM represents the quantitative MMP in each group. Values are expressed as the means ± SD. ^*^P<0.05 vs. control. ^#^P<0.05 vs. LPS/PepG group. n=3 per group.

JC-1 aggregates in the mitochondria emit red fluorescence (Fig. 4A and B, panel a). For a more objective understanding of MMP, confocal microscopy and FCM were both used to detect ΔΨm. Treatment of the H9C2 cells with LPS/PepG for 24 h re sulted in the dissipation of MMP, which was shown as increased green fluorescence (Fig. 4A and B, panel b) by JC-1 staining. To further elucidate the role of UCP2 in cardiomyocytes under septic conditions, the H9C2 cells pre-treated with LPS/PepG were transfected with ncRNA or siRNA. The red fluorescence intensity in the LPS/PepG group (Fig. 4A and B, panel b) was weaker than that in the LPS/PepG + siRNA group (Fig. 4A and B, panel d), but was similar to that in the LPS/PepG + ncRNA group (Fig. 4A and B, panel c). CCCP renders the mitochondrial innermembrane permeable to protons and causes the dissipation of the proton gradient across the inner mitochondrial membrane (Fig. 4A and B, panel e). To quantify the changes in MMP, the ratio of red fluorescence intensity to the green fluorescence intensity as shown by FCM was determined (Fig. 4C). In the control group, the ratio was 0.429±0.071, while the LPS/PepG-treated cells showed a lower ratio (0.222±0.038, P<0.05, vs. the control group, n=3) indicating the dissipation of ΔΨm in the H9C2 cells under septic conditions. The H9C2 cells transfected with LPS/PepG + siRNA demonstrated a rebound in the ΔΨm (0.563±0.121, P<0.05, vs. LPS/PepG group, n=3), while treatment with LPS/PepG + ncRNA had no effect on ΔΨm (0.219±0.197, P>0.05, vs. LPS/PepG group, n=3).

### Mitochondrial morphology and swelling in H9C2 cells under septic conditions

**Figure 5:**
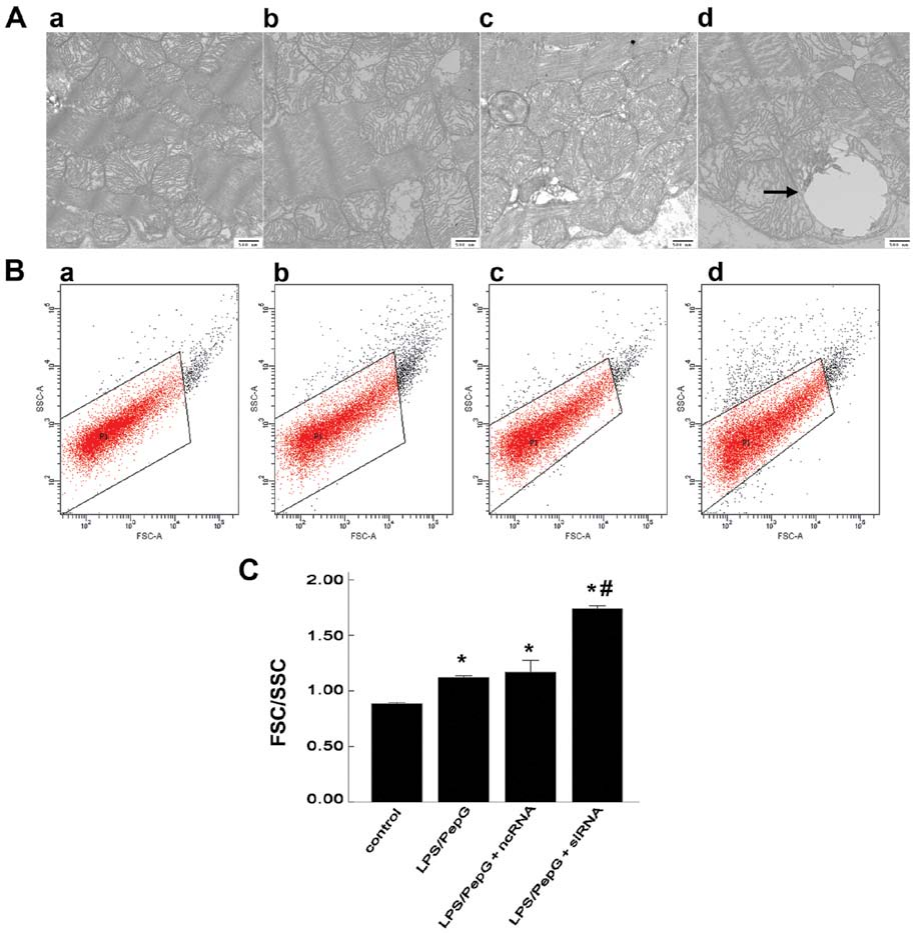
Effect of siRNA on mitochondrial morphology in H9C2 cells under septic conditions. (A) Scanning of H9C2 cell mitochondrial ultrastructure using a transmission electron microscope. Panel a, normal mitochondrial ultrastructure; panels b–d, swelling and vacuolization of mitochondria; panel d, megamitochondria (arrow); (original magnification, x30,000). (B) Flow cytometric determination of size [forward scatter (FSC)] and structure [side scatter (SSC)] of mitochondria in H9C2 cells. (A and B) Panels a–d, the control, LPS/PepG, LPS/PepG + negative control siRNA (ncRNA) and LPS/PepG + siRNA group, respectively (C) Quantitative evaluation of mitochondrial swelling by flow cytometry (FCM). The FSC/SSC ratio represents the degree of mitochondrial swelling. Values are expressed as the means ± SD. ^*^P<0.05 vs. control group. ^#^P<0.05 vs. lipopolysaccharide (LPS)/peptidoglycan G (PepG) group. n=3 per group.

In an aim to understand the mitochondrial structure more objectively, the mitochondrial ultrastructure was scanned by TEM and the quantitative degree of mitochondrial swelling was detected by FCM. Fifteen electron micrographs were prepared for each specimen and in each micrograph, 15 mitochondria were randomly selected. The ultrastructure of the mitochondria was normal in the control group, while mitochondrial swelling and vacuolization, loss of matrix and the disruption of crests were detected in the LPS/PepG-treated cells (with or without siRNA and ncRNA transfection). Compared with the LPS/PepG group, the mitochondrial ultrastructure was damaged more severely in the LPS/PepG + siRNA group in which megamitochondria were also frequently observed (Fig. 5A). Using FCM with appropriate settings, the FSC-SSC parameters were determined in the mitochondria. FSC highly correlates with cell size or volume and SSC is related to granularity and the refractive index (Fig. 5B). The quantitive degree of mitochondrial swelling was represented by the FSC/SSC ratio. The FSC/SSC ratio was 1.119±0.016 in the LPS/PepG group and 1.737±0.029 in the LPS/PepG + siRNA group. Both were markedly higher than that of the control group (0.882±0.012, P<0.05, n=3; Fig. 5C). Compared with the LPS/PepG group, treatment with LPS/PepG + siRNA induced a 1.6-fold increase in the FSC/SSC ratio (P<0.05, n=3), while LPS/PepG + ncRNA had no effect on this ratio (1.160±0.110, P>0.05, vs. LPS/PepG group, n=3; Fig. 5C).

### Effect of siRNA on intracellular ROS and ATP levels, and mtDNA copy numbers

**Figure 6:**
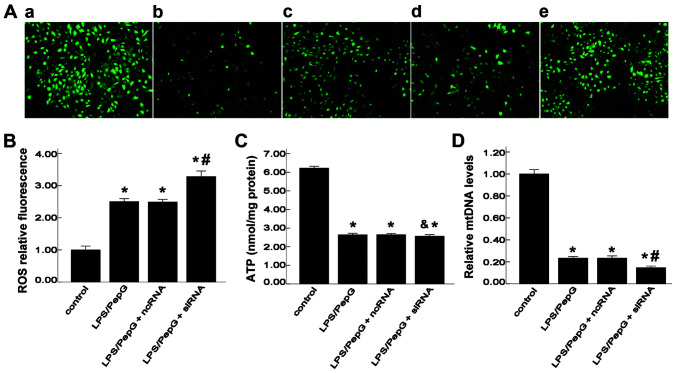
Effect of siRNA on intracellular reactive oxygen species (ROS) production, adenosine triphosphate (ATP) levels and mitochondrial DNA (mtDNA) copy numbers. The fluorescence intensity of DCF in H9C2 cells represented the production of intracellular ROS. (A) Changes in ROS production measured as the DCF fluorescence under a fluorescence microscope (magnification, x10); Panels a–e, ROS-positive control cells (Rosup), control, lipopolysaccharide (LPS)/peptidoglycan G (PepG), LPS/PepG + negative control siRNA (ncRNA) and LPS/PepG + siRNA group, respectively. (B) DCF fluorescence was read by a multifunctional microplate reader at an excitation wavelength of 488 nm and at an emission wavelength of 525 nm. (C) Cellular ATP expressed as nmol/mg protein was measured using a firefly luciferase-based ATP assay kit. RLU values were measured by a microplate reader. (D) Relative mtDNA copy numbers were detected by real-time PCR with normalization to 18s RNA. Values are expressed as the means ± SD. ^*^P<0.05 vs. control group. ^#^P<0.05, ^&^P>0.05 vs. LPS/PepG group. n=3 per group.

To elucidate the role of UCP2 in mitochondrial function, intracellular ROS and cellular ATP levels and mtDNA copy numbers were detected. DCFH-DA is a fluorescent probe of ROS. Intracellular ROS production was observed using a fluorescence microscope and quantified using a multifunctional microplate reader with DCFH-DA. The DCF fluorescence intensity was significantly higher in the LPS/PepG, LPS/PepG + ncRNA and LPS/PepG + siRNA groups than that in the control group (2.50±0.10, 2.49±0.08, 3.28±0.17, respectively vs. 1.00±0.12, F=183.8, P<0.05, n=3; Fig. 6A and B), indicating an enhanced production of ROS stimulated by LPS/PepG. The DCF fluorescence intensity in the LPS/PepG + siRNA group was notably increased compared with that in the LPS/PepG group (3.28±0.17 vs. 1.00±0.12, P<0.05, n=3; Fig. 6B). To further determine the effects of siRNA on mitochondrial function, indicators of mitochondrial activity, such as the levels of cellular ATP and mtDNA copy numbers were determined. After 24 h of exposure to LPS/PepG, the cellular ATP content was significantly decreased in the LPS/PepG, LPS/PepG + ncRNA and LPS/PepG + siRNA groups compared with the control group (2.643±0.076, 2.647±0.065, 2.560±0.092, respectively, vs. 6.220±0.105, F=1322.6, P<0.05, n=3; Fig. 6C). However, compared with the LPS/PepG group, ncRNA or siRNA had no effect on the ATP levels in the H9C2 cells under septic conditions (P>0.05; Fig. 6C). Cellular mtDNA is also a sensitive indicator of mitochondrial function. The mtDNA copy numbers in the LPS/PepG group were significantly lower than those of the control group (0.233±0.153 vs. 1.000±0.040, P<0.05, n=3; Fig. 6D). The mtDNA copy numbers in the LPS/PepG + siRNA group were significantly decreased compared with those in the LPS/PepG group (0.147±0.152 vs. 0.233±0.153, P<0.05, n=3; Fig. 6D). However, the mtDNA copy numbers in the LPS/PepG + ncRNA group did not differ from those in the LPS/PepG group (0.233±0.201 vs. 0.233±0.153, P>0.05, n=3; Fig. 6D).

## Discussion

The principal findings of the current study are as follows: i) treatment with LPS/PepG severely damaged the H9C2 cells and initiated an inflammatory response, indicating that the cell model of sepsis was successfully created; ii) siRNA down-regulated the expression of UCP2 at the transcriptional and translational level, indicating that UCP2 deficiency in the cells was successfully established; and iii) during sepsis, siRNA aggravated the injury to mitochondrial structure and eventually led to the dysfunction of the mitochondrion, demonstrating that UCP2 may play a protective role in the mitochondria in H9C2 cells under septic conditions. In brief, these findings support the hypothesis that UCP2 plays an important and protective role in cardiomyocytes under septic conditions.

This study demonstrates that the cell wall component, PepG, derived from the pathogenic Gram-positive bacterium, *Micrococcus luteus*, synergizes with LPS to damage H9C2 cells and causes the release of IL-6 and TNF-α *in vitro*. Sepsis is not only an unresolved problem in the medical field, but also a very serious threat to human health. Previous studies have created a number of models of sepsis *in vivo*, but few cellular models of sepsis have been created (23–25). LPS or LPS plus PepG are often used to induce sepsis in various cells which mimics models of sepsis *in vitro*. The significant increase in the levels of CK, LDH, TNF-α and IL-6 in our study indicated that the H9C2 cell model of sepsis had been created successfully. UCP2 is a member of the UCP family and is highly expressed in a number of tissues. Our previous study demonstrated that UCP2 expression was upregulated in the septic myocardium (unpublished data). To further determine whether UCP2 plays a protective role in the myocardium under septic conditions, we used siRNA to reduce the mRNA level of UCP2 by up to 69% in the current study. Our findings indicated that the silencing of UCP2 resulted in damage to the H9C2 cells, an increased release of TNF-α, the disruption of mitochondrial morphology, the rebound of MMP, enhanced ROS generation, as well as a reduction in cellular ATP levels and mtDNA copy numbers in the H9C2 cells under septic conditions.

MMP is dependent on the speed of proton pumping generated from the transport of electrons and protons. MMP reflects the performance of the electron transport chain (ETC) and this is often used as an indicator of the pathological disorder of the mitochondrion. As previously demonstrated, cells treated with LPS show a disruption of the MMP, which is a critical event in lethal cell damage (26,27). In this study, treatment with LPS/PepG induced the disruption of the MMP in the H9C2 cells and the silencing of UCP2 resulted in a marked rebound of MMP under septic conditions, which was consistent with the results of our previous study using a model of sepsis (unpublished data) and other models *in vitro* (27,28). The meaning of uncoupling is the collapse of the MMP by proton leakage through the mitochondrial membrane. UCPs, including UCP2 can disperse mitochondrial proton gradient to stabilize the inner MMP, so they are closely related to the MMP. For example, under MPP^+^ toxicity, UCP4 overexpression preserves ATP levels and MMP (29); the overexpression of UCP5 in neuronal cells can preserve the MMP, ATP levels and cell survival (30). Previous studies have demonstrated that MMP and ROS interact with each other (11,12,28).

Mitochondrial sources of ROS are considered as a basic cause of oxidant damage to the heart during sepsis, and they may underlie the pivotal mechanisms related to cardioprotection in the myocardium (31). Previous studies (32–34) have demonstrated that a slight degree of depolarization within the inner membrane of the mitochondria may play a protective role by attenuating ROS production. In the present study, the silencing of UCP2 by siRNA was associated with a relative increase in ROS production and a significant rebound in MMP during sepsis, which is consistent with uncoupling mechanisms (35). In UCP2 knockout mice, increased MMP production has been shown to cause vascular remodeling partially through increased ROS production (28). *In vivo*, previous neuronal studies have found that the overexpression of human UCP2 is associated with increased uncoupling and decreased ROS production, indicating that UCP2 plays a protective role (11,12,36). Our present finding that the silencing of UCP2 leads an increase in MMP and ROS production during sepsis suggests that UCP2 plays a protective effect in septic cardiomyocytes.

Compared with nDNA, mtDNA is more susceptible to oxidative damage, as the DNA repair capacity in the mitochondria is incomplete and mitochondria are an important source of ROS (37). ROS can oxidatively impair mitochondrial mtDNA (38,39) and trigger mtDNA deletions which can damage the synthesis of the mtDNA-encoded proteins of respiratory chain complexes I–V (40). In the current study, treatment with LPS/PepG resulted in damage to mtDNA and the overproduction of ROS which was not reversed by siUCP2, indicating that UCP2 may be a protective factor in cardiomyocytes under septic contitions. *In vivo*, LPS had been shown to cause mtDNA damage (41) which can activate the immune system and may contribute to SIRS and compromise organ function in a number of diseases (42–44). Research on the association between UCP2 and mtDNA in sepsis is limited, and the possible explanation of our finding is that the silencing of UCP2 through the alteration in ROS production, uncoupling activity and MMP, eventually leads to the deletion of mtDNA under septic conditions.

Apart from ROS and mtDNA, cellular ATP is another indicator of mitochondrial function. The mitochondria are the major source of ATP, which is the only universal energy-yielding currency in cells. ATP synthesis relies on the coupling of electron transport through the ETC to the proton motive force (45)

The coupling procedure is regulated by proton leakage through the mitochondrial inner membrane which is partly mediated by UCP2. However, the precise mechanisms through which UCP2 modulates this process continue to be a matter of debate. It has been demonstrated that UCP2 activity may lead to decreased ATP availability (46–49), although others believe that the overexpression of UCP2 results in elevated levels of tissue ATP (50–52). The association between UCP2 and ATP ramains debatable. In this study, sepsis damaged the H9C2 cells and led to low levels of ATP, while the silencing of UCP2 had no significant effect on the ATP levels (P>0.05). The alternative explanation may be that LPS/PepG is far more important than UCP2 in affecting the ATP level or UCP2 primarily has no effect on ATP in H9C2 cells under septic conditions.

To obtain more credible results, TEM was used for qualitative analysis and FCM was used for quantitative analysis. Our findings suggest that, under septic conditions, mitochondrial dysfunction increases oxidative stress, induces the collapse of the MMP and the deletion of mtDNA, which can, in turn, lead to mitochondrial swelling and damage to mitochondrial morphology. The silencing of UCP2 aggravated the degree of mitochondrial swelling and damage to mitochondrial morphology, indicating that UCP2 may be a protective factor in cells under septic conditions. There may be other explanations for the changes in mitochodrial swelling and morphology observed in this study. For example, the change in the Ca^2+^ concentration (53), intracellular ion levels (54), the status of mitochondrial pore (55,56), which regrettably were not detected in the current study; these may also affect the mitochondrial morphology and function. It is a pity that we only used siRNA for the study of UCP2 function in cardiomyocytes under septic conditions. To further clarify the association between UCP2 and mitochondrial function and to confirm the hypothesis that UCP2 may play a protective role during sepsis, the effects of the overexpression of UCP2 in H9C2 cells should be examined.

In brief, our study demonstrates that treatment with LPS/PepGn induces mitochondrial dysfunction in H9C2 cells and that the silencing of UCP2 by siRNA aggravates the damage to mitochondrial morphology and function, indicating that UCP2 may play a protective role in cardiomyocytes under septic conditions.

## Abbreviations

SIRS: systemic inflammatory response syndrome
SIMD: sepsis-induced myocardial dysfunction
ROS: reactive oxygen species
UCPs: uncoupling proteins
UCP2: uncoupling protein 2
siRNA: small interfering RNA
LPS: lipopolysaccharide
PepG: peptidoglycan G
ncRNA: negative control siRNA
LDH: lactate dehydrogenase
CK: creatine kinase
FCM: flow cytometry
TEM: transmission electron microscopy
MMP or ΔΨm: mitochondrial membrane potential
DCFH-DA: 2′,7′-dichlorofluorescein diacetate
DCFH: 2′,7′-dichlorofluorescein
mtDNA: mitochondrial DNA
nDNA: nuclear DNA
ETC: electron transport chain
JC-1: 5,5′,6,6′-tetrachloro-1,1′,3,3′-tetraethylbenzimidazole-carbocyanide iodine
